# Supplementary material for: Derivation of Highly Predictive 3D-QSAR Models for hERG Channel Blockers Based on the Quantum Artificial Neural Network Algorithm
Source: Pharmaceuticals (Basel). 2023 Oct 24;16(11):1509. doi: 10.3390/ph16111509 (PMC10675541; doi:10.3390/ph16111509)
Supplement: Supplementary file 1 [file pharmaceuticals-16-01509-s001.zip › pharmaceuticals-2656368-supplementary material.pdf]

Table S1. PubChem CID, molecular weights, experimental pIC_{50} values, and calculated pIC_{50} values of all the molecules in training sets.

PubChem CID	molecular weight	experimental pIC_{50}	calculated pIC_{50}
4893	383.41	4.62	4.91237
3386	309.33	4.91	5.09472
4212	444.49	5.45	5.50886
71329	441.56	8.39	8.31802
2913	287.40	4.77	4.89394
5775	281.36	4.70	4.86911
2160	277.41	4.75	4.75281
2267	381.90	7.00	7.23697
135398745	312.43	4.00	4.05538
3354	391.47	4.62	4.69784
3008902	512.69	6.26	6.26207
3008917	481.68	6.82	6.82021
56673962	555.76	6.28	6.18281
11619850	475.32	8.85	8.75532
11975438	380.42	7.70	7.52088
11539096	424.43	4.82	5.03626
91447871	417.52	5.28	5.12188
44417993	399.53	4.70	5.08565
11973802	437.47	4.32	4.13728
44417916	433.98	4.85	4.80751
44418482	339.48	3.99	3.74217

44418490	295.47	4.17	4.536
44418472	273.38	4.71	4.76457
44418491	287.40	3.93	3.74562
16220879	481.61	5.47	5.51511
44421703	388.53	5.85	5.39072
16220959	466.60	5.76	5.35944
16220791	418.56	5.29	5.32834
44421702	509.62	5.27	5.00865
16220874	515.07	5.59	5.53416
15986136	457.52	4.40	4.37202
23631000	527.61	7.20	7.157
23631092	494.05	7.10	7.20988
44438747	485.49	5.50	5.43784
44438750	571.59	5.07	5.21841
44438753	570.61	5.20	5.21979
44438755	550.63	5.18	5.13304
11713742	552.62	5.04	5.03554
44438745	551.63	6.13	5.88978
44438740	499.56	4.19	4.35669
44447783	556.59	7.17	7.15002
11585034	577.54	5.60	5.53094
11656848	548.65	5.90	5.7591
11496746	484.62	4.30	4.3722
11562113	483.58	5.90	6.00979

11669953	468.57	4.60	4.82717
44454278	454.55	7.20	7.31312
44454217	552.61	6.40	6.34725
44447775	558.58	7.35	7.38277
11554412	461.49	4.32	3.90378
11692376	540.73	5.80	6.12689
44454192	526.70	4.90	4.89665
11606456	554.75	5.40	5.43405
44454339	554.75	5.60	5.62133
44454340	526.70	4.40	4.32799
11712044	448.58	4.80	4.46972
44454248	577.54	5.60	5.52701
11519858	578.75	5.10	5.03803
11539722	452.57	5.20	5.08361
11527195	591.57	5.00	4.88313
85059382	335.40	4.56	4.54345
9883980	330.43	5.80	5.90079
44418471	299.42	4.55	4.54396
16220962	479.64	7.10	7.13597
11610716	385.51	5.06	5.14383
21973850	397.52	4.70	4.88984
44454275	563.51	5.20	5.16118
59050049	558.58	7.80	7.76412
85130389	558.56	6.07	6.06362

44593444	296.19	5.67	5.766
9814248	279.74	5.28	5.32721
44562616	300.15	5.91	5.90663
44562612	282.16	5.68	5.64594
9966330	307.17	5.34	5.00868
44563244	296.19	5.97	5.73463
44562611	296.19	5.75	5.9612
44563165	310.22	5.57	5.59893
44562614	300.15	5.68	5.90354
44562613	279.74	5.52	5.52314
9966438	310.22	5.83	5.6715
9966437	310.22	5.47	5.96293
75054235	517.71	6.61	6.60028
44563239	275.77	5.43	5.40315
44563241	275.77	5.28	5.39376
44563243	282.16	5.32	5.35229
44563238	282.16	6.19	6.12628
44143435	441.57	5.52	5.36342
44143456	461.02	5.47	5.27622
74933242	399.89	4.54	4.42038
44562944	295.30	4.98	5.09018
72996478	322.45	5.52	5.51031
11516387	377.49	5.30	5.32046
11675333	381.45	5.00	5.19201

75072777	411.49	5.92	5.7931
24901589	453.56	5.30	5.33267
10183035	460.51	4.87	4.82696
9919680	422.44	4.18	4.22559
74347291	405.36	6.03	5.82205
9944019	296.19	5.42	5.37285
74828503	574.49	5.92	5.71673
74828400	572.52	6.30	5.92905
57896619	586.55	7.40	7.48815
9947172	364.19	5.95	5.90156
9946374	350.16	5.92	5.8262
9966603	314.18	5.17	5.20878
74828378	550.51	6.05	5.80186
10115549	461.40	6.77	6.71997
25107622	416.89	5.72	6.01125
10143631	541.46	5.92	5.8715
44560681	554.65	5.00	5.08658
74932521	387.42	5.20	5.48482
43314375	293.76	5.34	5.41002
10132572	350.16	5.88	5.72317
44143413	434.79	7.64	7.64528
44563240	289.80	5.61	5.71957
23151331	381.45	5.00	5.00617
9817349	286.34	5.70	5.57962

9979961	427.49	4.72	5.11308
74347296	350.50	4.96	4.67631
74828379	586.49	5.60	5.51392
57896613	573.51	6.00	5.9068
15986914	435.57	4.50	4.50298
15986644	467.99	4.50	4.57376
56667460	383.55	7.96	7.85846
56664290	350.46	6.12	6.36103
45272411	388.47	5.00	4.9214
45270870	445.57	5.32	5.34592
45272437	346.47	4.20	4.18525
56660415	550.18	5.89	6.00692
45271510	321.85	5.80	5.76712
75107798	423.98	7.20	7.1667
45272414	389.42	5.20	5.36004
45272426	321.85	5.50	5.30464
56674400	318.48	5.64	5.43581
56663998	442.62	5.70	5.76462
45271760	399.93	5.80	5.95162
45270796	355.44	5.37	5.20192
45273266	359.83	5.50	5.68622
56678014	318.48	5.12	5.14124
56677672	344.47	5.11	5.17213
56667448	525.73	5.02	4.98928

24749507	421.54	4.20	4.2185
45269119	358.44	5.16	5.11396
56674421	462.62	5.02	5.20611
44517726	341.84	5.50	5.52466
56664310	442.62	5.93	5.99458
56670771	427.95	4.82	4.96964
45269118	402.49	6.04	6.10706
56671428	393.93	4.80	5.01396
45268438	295.81	5.50	5.67065
45273293	359.45	4.80	4.74768
45268127	322.41	4.50	4.61095
45272425	403.45	5.90	5.7781
75107919	375.94	5.90	5.82211
45487513	351.49	5.20	5.1713
45482705	394.54	4.71	5.00792
45482753	436.57	5.07	5.21205
45482728	395.52	3.72	3.67683
44627023	451.63	6.00	5.85775
44627357	481.51	6.20	6.21426
44626639	534.52	6.30	6.24275
24743758	481.51	6.00	6.04577
44626632	455.62	5.30	5.21521
67365726	460.52	5.40	5.43525
44627025	474.42	6.10	5.88607

75228257	348.45	4.65	4.55745
25156194	331.46	4.58	4.53645
75228342	335.45	4.40	4.59841
75228283	317.43	4.55	4.39735
87964253	351.87	5.44	5.30039
11696460	374.48	5.44	5.37828
75228322	320.44	4.75	4.72143
44216386	317.43	4.92	5.05816
25156476	306.41	4.40	4.58052
21973860	399.58	5.55	5.57465
85120740	325.47	4.60	4.68405
75228298	254.37	3.66	3.93772
21973907	404.53	5.55	5.58615
46890443	350.38	4.98	4.99121
25156477	386.29	6.06	6.1505
46884462	389.90	5.18	5.11194
46883917	453.94	4.92	4.99324
46888778	555.88	5.80	5.83017
46888749	541.85	5.28	5.2949
46888750	555.88	5.06	5.09984
46888366	577.89	5.34	5.24972
46888748	555.88	5.92	5.89687
46888776	527.83	6.42	6.86846
52940753	477.55	6.70	6.56416

52949300	521.66	6.20	6.27734
24758715	488.44	6.10	5.98916
24758947	474.08	5.60	5.62849
52946432	427.54	5.60	5.6365
68913061	364.43	4.90	5.14048
25072568	446.43	4.30	4.21692
25072244	450.39	4.80	4.86703
25022356	348.40	6.00	6.03697
73023748	359.49	4.50	4.39501
71851	265.44	5.23	5.21398
11502008	390.48	8.09	8.13052
72968146	523.69	5.30	5.34771
75953517	509.04	7.18	7.02696
76144979	473.01	5.08	5.00732
25151793	545.60	5.62	5.61326
25151988	491.63	4.95	4.93857
25153077	582.62	5.92	5.80298
74072623	508.62	5.89	5.78928
76152437	499.57	5.12	5.11127
76152796	500.56	5.05	4.97919
25125138	503.20	7.00	7.14261
56659871	427.46	5.09	5.10295
56659872	401.49	5.67	5.6014
73687875	391.51	5.13	5.27337

56666240	306.41	5.08	4.98254
56673157	322.47	5.32	5.25201
56673090	377.91	5.70	5.92166
56659225	419.49	5.00	5.09482
56664483	442.62	5.72	5.63648
56670257	349.52	5.09	5.13516
56683751	295.42	6.38	6.35939
46241829	476.50	4.46	4.46074
46947816	476.50	5.00	5.02799
56675301	439.48	5.35	5.3683
54755801	559.59	5.12	4.91045
76391939	326.40	5.60	5.77562
54756045	510.51	4.70	4.65943
56678506	545.57	4.60	4.53375
56657930	528.62	4.92	4.77154
56671794	516.56	4.92	5.02074
56683044	521.56	4.51	4.48521
56676480	298.35	5.80	5.80474
76389284	326.40	5.60	5.51485
11660134	340.43	5.20	5.2511
76391512	342.40	5.40	5.55273
76393702	342.40	5.10	5.16509
76392272	328.37	5.40	5.56027
76392996	376.41	5.50	5.36633

10324551	452.53	5.30	5.27612
53372672	381.48	4.50	4.40117
57663666	439.49	4.70	4.59266
57663581	438.50	4.20	4.13996
24782303	441.48	5.10	4.956
76586948	435.50	5.20	5.36196
57663596	439.49	4.20	4.17932
57663447	425.57	4.60	4.45917
24785870	397.52	4.60	4.49996
34598448	284.37	4.62	4.6932
60145493	312.43	4.87	4.78437
57803392	567.61	4.52	4.73187
57803351	595.67	5.00	5.01298
44332931	396.53	7.11	7.4415
78073551	369.85	5.90	5.93606
71449253	361.45	7.38	7.43143
71456366	364.45	7.66	7.64425
71451051	471.55	6.82	6.77844
71461788	357.43	7.64	7.54793
66615614	503.05	4.50	4.34836
46241611	506.52	4.76	4.88239
46241717	520.55	4.84	4.74666
46240584	532.58	5.21	5.20833
91248210	508.37	5.70	5.65369

78074841	453.36	5.60	5.81296
91282137	568.46	5.20	5.00993
68003782	506.54	4.55	4.82457
68004010	548.62	4.43	4.17992
68004011	530.55	4.54	4.62682
71552097	499.54	4.34	4.3756
71552261	573.55	4.44	4.27011
71604552	362.49	6.68	6.7864
71604556	431.37	7.85	7.91257
12429371	287.40	5.79	5.7969
71605712	334.44	7.28	7.21609
71606402	438.59	4.84	5.01792
71606877	330.49	7.62	7.7013
71606712	310.50	5.70	5.74148
71604701	318.56	7.82	7.89128
57609886	294.38	6.26	6.31141
71606230	306.47	5.36	5.40329
71606232	446.57	6.53	6.32159
72375832	500.48	5.29	5.22211
72375835	500.48	5.16	4.92679
69087562	466.92	4.77	5.06387
69087440	446.51	5.00	4.95953
57331240	421.46	6.80	6.74451
72375747	418.50	5.60	5.5517

72793448	335.49	5.32	5.31509
72793446	293.83	5.13	5.36984
72793578	279.85	5.73	5.63731
72793579	321.93	6.62	6.62083
72793520	273.46	5.93	6.08342
12951762	275.48	6.30	6.24164
72793582	281.86	5.60	5.52463
72793583	323.95	6.91	7.23016
72793584	287.44	6.91	6.90003
72793304	291.86	5.39	5.23132
72793308	271.44	5.45	5.60649
72793309	263.81	5.58	5.66743
72793378	305.89	6.06	6.02772
72793380	285.47	5.29	5.3065
72793383	285.47	5.36	5.27448
72793384	277.83	5.22	5.17444
72793441	299.50	5.72	5.76216
72793442	273.80	5.00	4.92698
76335400	279.81	4.84	4.90303
76331825	321.89	5.61	5.28639
72793517	265.82	5.95	6.03021
45278564	486.44	5.77	5.88438
90667423	554.57	4.06	4.15687
90667586	544.53	6.41	6.23542

90667590	442.48	4.03	3.68281
90667600	486.54	4.09	3.97576
68154116	261.72	4.30	4.45644
118707596	260.29	4.68	4.52053
118707597	276.74	4.68	4.90961
118707602	276.74	4.22	4.64974
118707603	262.71	4.70	4.68584
118710536	533.56	4.02	4.21893
118735868	505.57	5.84	5.75266
118735870	553.63	5.81	5.89549
71152709	521.55	8.06	7.9086
118736035	555.62	7.91	7.9167
71153161	495.54	8.78	8.90763
118736044	505.57	8.38	8.41192
122179455	398.51	6.47	6.45422
122179465	509.54	5.37	5.54359
122179473	497.49	4.67	4.82304
122179478	539.57	5.11	5.10408
122179483	568.57	4.46	4.40054
122182831	422.46	4.66	4.76808
122182832	404.47	4.59	4.44669
122182834	404.47	5.66	5.51449
122182881	421.50	4.41	4.08793
71153095	527.98	5.24	5.16226

71181567	511.53	5.89	6.08263
71487141	533.60	6.30	6.20009
122186032	579.63	5.44	5.45785
71152895	507.56	6.10	6.07385
78023056	591.64	6.22	6.22459
71153418	564.61	4.61	4.62796
71153030	546.60	5.05	5.22064
71181641	558.61	5.26	5.11273
71153157	593.65	6.00	6.02449
71153063	593.65	6.40	6.49292
71152932	537.59	5.62	5.61338
71152803	551.62	5.62	5.64373
122186271	567.62	4.98	5.11896
71153155	562.64	5.12	5.52571
71152823	548.62	5.43	5.5077
59472603	478.50	5.05	4.98599
59472511	468.56	5.80	5.90412
59472517	514.48	5.57	5.36098
122187730	510.64	5.05	4.93772
67516635	433.55	5.89	5.89694
71535563	359.81	4.58	4.88883
71152655	537.55	6.00	5.98817
127036551	566.59	4.74	4.66668
71181421	553.59	5.85	5.87816

90355676	577.53	5.62	5.52548
71152955	551.53	4.95	5.00448
127037414	507.52	5.92	5.80981
71152850	507.52	5.03	5.19601
127034022	333.47	5.82	5.77278
11544795	348.49	6.03	5.96014
127034292	338.47	6.70	6.71479
11624970	374.52	6.30	6.34479
127027566	479.49	4.72	4.64692
69087452	461.52	4.28	4.35812
127028816	475.55	4.38	4.021
76747503 (chemspider ID)	392.41	5.65	5.71026
134146922	452.46	4.98	5.01787
118864337	308.26	3.86	4.25411
137637204	597.57	4.85	4.66486
121483470	475.55	5.48	5.59467
25189510	461.66	7.02	6.89245
137631897	285.27	5.05	5.29046
130449717	441.54	5.10	4.85564
121344565	452.51	4.67	4.62713
118334979	353.36	5.70	5.61052
78041485	571.53	5.35	5.37517
137651081	446.50	5.75	5.69691
137645234	321.25	4.82	4.89236

121314002	438.53	5.35	5.15678
65459349	285.27	5.75	5.87709
137647028	457.40	5.00	5.20633
137647807	544.64	4.85	4.75863
25190134	404.57	5.78	5.86871
67264950	337.42	4.66	4.67486
137643261	437.54	5.48	5.40011
71223050	569.54	5.24	5.13787

Table S2. PubChem CID, molecular weights, experimental pIC_{50} values, and calculated pIC_{50} values of all the molecules in test sets.

PubChem CID	molecular weight	experimental pIC_{50}	calculated pIC_{50}
3100	255.36	4.50	6.22323
44417919	433.98	5.58	9.17927
11973800	398.41	7.67	3.72536
11569300	485.33	8.60	7.35554
44418497	347.46	3.93	4.00808
11663999	551.75	5.90	7.01757
23630796	524.68	6.10	8.58192
11692293	534.63	4.18	5.73231
44442481	386.42	5.58	8.19453
11649549	539.62	4.38	9.36729
85051858	542.56	5.75	6.47375
11569996	525.71	6.00	6.057

11613873	566.64	5.60	5.62079
11548755	580.67	6.60	7.14462
11497680	552.61	5.70	9.53744
44418473	271.36	4.28	7.47262
15987451	443.49	5.10	7.62239
44442488	398.48	5.00	7.52042
44417895	513.65	5.30	6.17241
74983959	414.54	5.36	8.7677
9835490	283.70	5.08	5.87855
15979148	577.69	5.56	8.42995
44563206	310.22	5.94	5.53712
44589077	400.27	4.50	5.96448
24948948	381.45	5.22	4.31727
43314210	259.32	4.70	4.94574
9965169	265.71	5.13	6.0354
44562989	282.16	5.41	3.34054
25107759	489.45	6.14	4.69235
9879772	312.19	6.16	2.8279
44563204	315.72	5.29	7.68583
44563203	326.22	6.11	4.21917
56670908	599.73	5.13	8.80686
56677638	560.17	5.68	5.64702
56677612	296.37	5.04	4.76573
75108057	309.79	5.30	2.45035

56670755	338.45	5.97	7.99489
45270903	347.88	6.40	8.10107
45271511	357.37	4.80	4.78173
72976566	473.56	6.10	9.3539
85146495	345.49	4.70	1.45229
18452955	377.51	5.55	6.2589
85134103	351.51	4.69	9.71113
85120052	280.41	4.36	6.793
21973904	433.50	5.55	7.80069
21973886	399.55	5.87	6.11901
46888688	573.82	6.22	4.89441
69255825	479.65	5.20	2.56501
69256095	513.67	5.89	4.87403
53317307	440.54	9.17	7.54452
11596311	392.50	8.01	0.95638
75596930	517.65	8.00	9.08813
76153126	512.61	4.77	5.08317
73687874	411.50	5.83	2.71404
56673158	322.47	5.61	3.98728
46946452	498.55	5.62	0.48518
56664979	529.56	6.08	7.23569
56666237	340.43	5.60	5.03763
24785864	441.48	5.10	4.18679
24785333	455.49	4.80	7.97115

70685675	298.40	4.03	6.10449
78070900	368.86	5.90	5.10301
78072941	410.94	8.40	8.00826
56970864	431.53	6.33	8.87946
68154164	259.30	4.49	6.51082
69274365	477.42	4.50	9.14253
46240482	482.52	4.50	5.66272
91410354	597.51	6.00	5.24899
71720519	283.41	8.29	2.71066
71606398	363.26	5.41	6.21416
69087495	446.51	4.70	9.11345
58576126	273.46	7.24	5.54435
72793514	293.88	6.61	3.60455
118707606	261.27	4.39	6.34382
71152722	488.52	8.41	7.60934
118736041	493.54	8.60	7.37656
71152755	506.58	8.92	5.76261
118736776	462.52	7.19	8.25625
122179469	541.60	5.80	6.39264
122179482	569.55	4.52	6.85394
71153175	579.63	4.36	3.68681
78015474	577.61	4.40	4.58702
71152975	565.64	6.16	9.0444
71152643	550.63	5.10	8.02864

71536660	386.88	4.91	5.84627
71152656	523.56	5.21	7.46987
89315867	522.53	5.27	5.11206
86730519	447.49	5.19	8.77993
118864285	338.74	5.09	7.11262
56603301	474.48	5.11	8.27373
89464128	336.35	4.40	6.51214
25190431	461.66	6.60	5.79501
137631834	315.29	5.43	7.24104
137656215	378.39	4.88	4.60133
56603116	472.51	5.27	8.4761
